# Identification of Heat Tolerant Cotton Lines Showing Genetic Variation in Cell Membrane Thermostability, Stomata, and Trichome Size and Its Effect on Yield and Fiber Quality Traits

**DOI:** 10.3389/fpls.2021.804315

**Published:** 2022-01-05

**Authors:** Saifullah Abro, Muhammad Rizwan, Zaheer Ahmed Deho, Shafiq Ahmed Abro, Mahboob Ali Sial

**Affiliations:** ^1^Plant Breeding and Genetics Division, Nuclear Institute of Agriculture (NIA), Tando Jam, Pakistan; ^2^Technical Services Division, Nuclear Institute of Agriculture (NIA), Tando Jam, Pakistan

**Keywords:** breeding, cotton, high temperature, germplasm, physiology, stress tolerance

## Abstract

Heat stress in cotton reduces its productivity. The development of heat-tolerant cotton varieties having resilience against changing climate is feasible. The purpose of this study was to probe the genetic variability in upland cotton for heat tolerance, the association of cell membrane thermostability (CMT), stomata, and trichome size with cotton adaptation to high temperature and effective breeding strategy to advance the valued traits. Relative cell injury percentage (RCI%) in studied genotypes ranged from 39 to 86%. Seventeen genotypes were found heat tolerant on the basis of low RCI%, heat susceptibility index (HSI<1), higher number of boll/plant, and seed cotton yield (SCY). Scanning electron microscopy (SEM) of heat-tolerant genotypes revealed the presence of different size of stomata (21.57 to 105.04 μm^2^) and trichomes (177 to 782.6 μm) on leaves of selected genotypes. The regression analysis showed a strong and negative association of RCI% and stomata size with SCY. However, no association was observed between the trichome size, yield, and fiber traits. On the overall location basis, a significant genotype × environment interaction was observed. All selected genotypes produced a higher SCY as compared with check varieties. But the stability analysis showed that the high yielding genotypes NIA-M-30, NIA-80, NIA-83, and CRIS-342 were also wide adaptive with unit regression (bi∼1) and non-significant deviation from the regression line (S^2^d∼0). The ability for the combination of some heat-tolerant genotypes was estimated by using the line × tester method among nine hybrids along with their 3 testers (i.e., male) and 3 lines (i.e., females). Genotypes, CRIS-342 and NIA-Perkh, were observed as best general combiners for SCY with a negative general combining ability effects for RCI%. Five hybrids showed a positive specific combining ability and heterotic effects for studied traits and also found lowest for HSI. RCI% and SCY/plant displayed higher estimates of heritability and genetic advance, indicating the heritability due to additive gene effects and chances of effective selection. The identified heat-tolerant and wide adaptive germplasm can be further advanced and utilized in cotton breeding programs for developing heat-tolerant cultivars. Selection criteria involving CMT and stomata size concluded to be an effective strategy for the screening of heat-tolerant cotton.

## Introduction

Cotton (*Gossypium hirsutum* L.) is an international agricultural product of which quality and quantity are subject to various whims of nature. It occupies an important position in global status of commercial crops with annual impact of >US$50 billion in the economy of the world (Organisation for Economic Co-operation Development/Food and Agriculture Organization [OECD/FAO], 2019). The lint quality in general, while the quantity of produce, i.e., the seed cotton yield (SCY) particularly is highly sensitive to climatic conditions. It can be seen in case of Pakistan where it was grown across 2.3 million hectares during 2018–2019 with an average per hectare yield of approximately 707 kg/ha (Anonymous, 2018–2019)) compared with 2,320 and 1,765 kg/ha for Australia and China, respectively (International Cotton Advisory Committee [ICAC], 2018). The quality of lint produced is also inferior, having a short fiber length, coarse fiber fineness, and lower uniformity, resulting in a higher import of longer fiber and lower price of locally produced cotton lint (Pakistan Central Cotton Committee [PCCC], 2014). The cotton belt of Pakistan is mainly located in the high-temperature zone where mean maximum temperature often exceed 48°C during the cotton-growing season. The optimum temperature for a successful cotton production is about 35°C, which may vary among cultivars. However, temperatures above this threshold level badly affect the peak time of cotton flowers and boll setting, resulting in excessive evapo-transpiration and abscission/no boll set at lower half of the plant (Bibi et al., 2003). Singh et al. (2007) reported a strong negative correlation between high temperature and the lint yield, resulting in a yield decrease of about 110 kg/ha for each unit increase in maximum temperature.

Identification of genotypes that have a greater ability to withstand the peaks of heat stress coupled with their limited water use is important to enhance the cotton productivity (Cottee et al., 2010). However, suitable selection standards are required for measuring resilience of cotton germplasm against heat stress. Plant phenological traits especially flowering and boll retention capacity in high temperature environments are effective in repeatable heat stress screening environments (Ismail and Hall, 1999). But genotypes with significant G × Y (genotype × year) and G × E (genotype × environment) interaction in the field often compromise genetic advance. Therefore, genetic gain in the cotton yield can be exploited by targeting those traits that are closely associated with plant adaptation to high-temperature environment (Singh et al., 2007). Cell membrane thermostability (CMT) has been reported as most relevant and suitable selection criteria for measuring heat tolerance (Abro et al., 2015). The ratio of trichomes to stomata is also found associated with the efficiency of water use (Galdon-Armero et al., 2018; Lei et al., 2018). Slow progress in development of heat tolerance in cotton cultivars due to its polygenic inheritance has been reported in earlier findings (Jones et al., 2014). A low heritability and time reliance of adaptive traits in high-temperature environments have also been reported as major obstacles (Singh et al., 2007; Jones et al., 2014). However, combining morphological and plant physiological traits for the elucidation of thermo tolerance in cotton is important and expected to result in improved heat tolerance in cotton (Rana et al., 2011). Better understanding of genetic, morphological, and physiological traits conferring heat tolerance in cotton is necessary, if climate change resilience, high yielding, and fine fiber quality of cotton cultivars are to be developed. An ability for the combination of genotypes in general (general combining ability [GCA]) and specific combining ability (SCA), heterotic effects and heritability of the traits coupled with genetic advance are also important in devising an effective breeding strategy for improving heat stress tolerance of cotton genotypes (Azhar et al., 2005).

The present study investigates the genetic variability in upland cotton for heat tolerance and identifies the association of CMT, stomata, and trichome size with cotton adaptation to high temperature and effects on yield and fiber quality parameters. The line × tester analysis was also performed for deciding the effective breeding strategy to advance the valued traits.

## Materials and Methods

### Study Site and Experimental Material

The research work was conducted at Plant Breeding and Genetics Division, Nuclear Institute of Agriculture (NIA), Tando Jam, Pakistan, between the years 2015 and 2019. The experimental material consisted of fifty-eight diverse cotton genotypes including advance lines developed at NIA using hybridization and radiation-induced mutagenesis. History of breeding material used in the present study is given in Supplementary Table 1.

### Evaluation of Cotton Genotypes for Heat Stress Tolerance

Heat tolerance of the experimental material in terms of high CMT, boll retention, SCY, ginning out turn percentage (GOT%), and staple length was tested in two sowing dates (i.e., early and normal) in a randomized complete block design (RCBD) with three replications. The RCBD was used to evaluate the large set of 58 genotypes because of the non-significant within-block variability and also because we were using the same piece of land every season for cotton breeding trials only. Further, the plot size used was smaller than the normal plot size being used for advance line yield trials. One set of experimental material was sown early (15 March, 2015) to expose the genotypes to high temperature (up to 45°C in the months June and July) at the peak flowering and boll setting stage. Another set of same genotypes was planted in normal sowing (15 May, 2015) to experience mild temperature at the flowering and boll setting stage during late August. Cultivars CRIS-134 and CIM-469 were included as heat tolerant and susceptible check, respectively. Each genotype was planted in a plot size of 6.1 × 3.0 m in four rows with 30 cm plant-to-plant and 75 cm row-to-row distance. Standard agronomic and plant protection measures were performed throughout the growing season. From each genotype, five random plants were selected for data recording on number of bolls/plant. At maturity, seed cotton was picked, and the data were recorded on SCY. Further, seed cotton from each repeat of each genotype was ginned using the ginning machine. The staple length (in mm) was measured using the fibrograph by taking forty grams of lint from each sample. GOT% was calculated by following formula.


$$\mathrm{GOT}\%=\frac{\mathrm{Lint}\,\mathrm{weight}}{\mathrm{Weight}\,\mathrm{of}\,\mathrm{seed}\,\mathrm{cotton}}\times 100$$

### Analysis of Cell Membrane Thermostability

Relative cell injury percentage (RCI%), a measurement for CMT under a high temperature stress, was determined according to Sullivan (1972). RCI% was measured from fully developed green leaves of 20–22 days during the peak flowering season. A steel punch with a 10 mm inner diameter was used for collection of two sets of leaf disc from both sides of the midrib of selected leaves. One set was used for heat treatment, and other was kept as control. Leaf discs were collected at about 1 pm to 3 pm, and samples were immediately kept in glass vials having 2 ml of deionized water. Leaf discs were thoroughly washed two times with deionized water to rinse any electrolyte adhering on the outside. After the final rinse, 2 ml of deionized water was added in sterilized glass vials and covered with a lid to avoid evaporation during heat treatment. One set of glass vials was kept at room temperature and other at 50°C in a water bath for 1 h. After heat treatment, 10 ml of deionized water was added in vials and kept at 10°C for 24 h to allow diffusion of electrolytes. On the next day, vials were kept at room temperature and shaken three times for mixing of electrolytes. Initial electrical conductivity (EC) was measured using the EC meter (Model HI-933300, Hanna Instrument, United States). After the measurement of EC, the samples were autoclaved for 10 min at 0.10 MPa pressure for releasing all electrolytes. The same procedure for measurement of EC was repeated. RCI% was calculated by using the following formula:

RCI% = 1-[{1-(T1/T2)}/{1-(C1/C2)}] × 100

Where as

T1 and T2 = EC of sap at 50°C before and after autoclaving, respectively.

C1 and C2 = EC of sap at 25°C before and after autoclaving, respectively.

### Measurement of Heat Susceptibility Index

Heat susceptibility index (HSI), a measurement of a relative decrease in yield influenced by non-favorable vs. favorable environments, was determined according to Fischer and Maurer (1978).

Heat susceptibility index = [(1-yh/yp)/H]

Where:

Yh = Average yield of genotypes under heat stress

Yp = Average yield of genotypes under non-stress condition or potential yield

H = Heat stress intensity = 1-(average yh of all genotypes/average yp of all genotypes).

### Scanning Electron Microscopy of Leaves for Stomata and Trichome Size

For scanning electron microscopy (SEM) study of selected heat-tolerant genotypes along with the susceptible check, prepared leaf specimens were mounted on the stubs of a double-sided cellophane tape. The sputter was covered with Jeol, JFC-1500 ion Sputter device with C-30–50 mm gold. The sample specimens were observed, and pictures were taken by SEM, Jeol, JSM T-200, and Jeol-T6380 at voltage of 5–15 KV with a distinct magnification (Hayat, 1989). The stomata and trichome measurements with 4–5 repetitions were carried from an individual leaf using an SEM measuring bar. The SEM study was conducted at Central Research Laboratory, University of Karachi, Pakistan.

### Multienvironment Field Trial of Selected Heat-Tolerant Genotypes for the Stability Analysis

Seventeen selected heat-tolerant genotypes along with one susceptible check (CIM-469) were evaluated for their stability analysis across five different locations of Sindh (i.e., Tando Jam, Matiari, Shaheed Benazirabad, Dadu, and Khairpur) during 2016–2017. The multienvironment trials were conducted in the RCBD with four replications. The plot size was 18 m^2^ (6 × 3 m) with a row-to-row and plant-to-plant distance of 75 and 30 cm, respectively. Standard agronomic and plant protection measures were adopted throughout the growing season. At maturity, seed cotton was picked, and data were recorded on SCY (kg/plot).

### Population Development for Genetic Studies of Heat Tolerance

To determine the pattern of inheritance and genetic variability, three heat-tolerant cotton genotypes (i.e., CRIS-342, NIA-Perkh, and CRIS-134) and three heat-susceptible genotypes (i.e., HariDost, Sohni, and NIA-148) were selected as parents for population development. The genetic population was developed by following the line × tester (3 × 3) method. Heat-tolerant genotypes were used as testers (i.e., male), whereas heat-susceptible genotypes were kept as lines (i.e., female). For crossing, unopened flowers commonly known as candles or buds were hand emasculated in the evening and covered with a butter paper bag. Male parent flowers were also covered with butter paper bags to avoid insect-intervened pollen contamination. Pollination of flowers was carried out in the morning (10:00 AM), and pollinated flowers were again covered with butter paper bags. At maturity, seed cotton was picked from all cross combinations. Ginning was performed, and F_0_ seeds were collected for evaluation in F_1_ generation along with parents.

### Evaluation of F_1_ and F_2_ Generations

Two sets of F_1_ genetic material (i.e., 9 hybrids) along with six parents were planted in the field (i.e., normal) and glasshouse (i.e., elevated temperature) in the RCBD with 3 replications. The data were recorded on five random plants for RCI% and SCY (g/plant) in each cross and analyzed for combining ability and heterotic effects. Seeds harvested from F_1_ generation were again evaluated in F_2_ generation along with parents. Again two sets were planted, one in the field (i.e., normal) and other in the glasshouse (i.e., elevated temperature) conditions. In F_2_, 10 and 20 plants were maintained in each cross under the glasshouse and field experiment, respectively. The data on five random plants in each cross along with parents were recorded for RCI% and SCY (g/plant). Genetic variance, heritability, and genetic advance were estimated from F_2_ data.

### Statistical Analysis

The data collected were analyzed separately for each parameter and subjected to analysis of variance (ANOVA) following Steel et al. (1997). The means comparison (honest significant difference [HSD] test at alpha 0.05) and correlation matrix were computed using the STATISTIX^®^ VERSION 8.1 software. The values presented are mean of three replicates ± standard error (SE). The stability analysis of heat-tolerant genotypes was carried out for SCY data from replicated trials at multienvironments. The analysis comprised location-wise or environmental ANOVA and the combined ANOVA for any place/environment (Pooled ANOVA). Stability parameters were calculated following the model by Eberhart and Russell (1966). For the combining analysis, the mean squares of the line × tester design, the overall ability of the combination (i.e., GCA), and SCA variances and effects were calculated according to the method proposed by Kempthorne (1957). Mid-parent heterosis and better parent heterosis were worked out as percent mean deviation of the mean F1 performance over the mean performance of the mid-parent and better parent, respectively. Genetic variance was calculated by following Singh and Chaudhary (1999). Heritability was calculated by following Cochran and Cox (1957), and genetic advance was estimated according to Allard (1960).

## Results

### Response of Different Genotypes of Cotton to Heat Stress

The analysis of variance showed highly significant differences among studied genotypes for RCI%, SCY, and number of bolls/plant. Differences were also observed among sowing dates for number of bolls/plant and SCY. Significant differences were present among sowing dates for RCI%, staple length, and GOT%. The sowing dates × genotypes interaction was also found highly significant for number of bolls/plant and SCY (Supplementary Table 2). The RCI% values of 58 genotypes are presented in Table 1. RCI% in studied genotypes ranged from 39 to 86%. The RCI level was noted >70% in 22 genotypes, while 60–70% in 20 genotypes and 50–60% in 08 genotypes. Eight genotypes showed < 50% level of cell damage, i.e., 40–50%. The tolerant genotypes, 50–60% levels of cell damage, were Sadori, NIA-Perkh, NIAB-111, CRIS-134, and CRIS-342. Whereas, some advance lines such as NIA-80, NIA-82, NIA-83, NIA-84, NIA-Bt1, NIA-Bt2, NIA-M-30, NIA-M31, NIA-HM-48, NIA-H-32, NIA-HM-327, and NIA-HM-2-1 had the lowest RCI level < 50% (i.e., higher CMT). The genotypes NIA-86, NIA-M2, NIA-M32, NIA-HM-1, NIA-H67, CRIS-121, and CIM-496 showed the highest RCI level (i.e., lower CMT). Out of 58, 17 genotypes were identified as more heat-tolerant because of having a higher CMT.

**TABLE 1:** Mean performance of 58 cotton genotypes for relative cell injury percentage (RCI%), yield, and fiber traits.

Genotypes	RCI%		No. of bolls/plant		Seed cotton yield (Kg/ha)		Staple length (mm)		GOT%	
	SD 1	SD 2	SD 1	SD 2	SD 1	SD 2	SD 1	SD2	SD 1	SD 2
NIA-80	45 ± 1.7	42 ± 1.2	39 ± 0.1	41 ± 0.1	3871 ± 7	3912 ± 7	28.0 ± 0.3	28.5 ± 0.1	36.0 ± 0.6	37.5 ± 0.3
NIA-81	57 ± 0.6	55 ± 0.6	35 ± 0.1	37 ± 0.1	3669 ± 6	3732 ± 18	28.0 ± 0.3	28.5 ± 0.1	38.5 ± 0.3	39.4 ± 0.3
NIA-83	55 ± 0.6	53 ± 0.6	35 ± 0.1	38 ± 0.1	3584 ± 2	3610 ± 6	27.8 ± 0.1	28.1 ± 0.1	36.0 ± 0.3	37.0 ± 0.6
NIA-84	57 ± 0.6	55 ± 0.6	38 ± 0.1	39 ± 0.0	3678 ± 6	3710 ± 6	28.2 ± 0.1	28.5 ± 0.2	36.5 ± 0.3	38.4 ± 0.2
NIA-85	78 ± 0.6	73 ± 1.6	31 ± 0.1	36 ± 0.0	2415 ± 9	2934 ± 12	27.0 ± 0.0	28.0 ± 0.0	34.5 ± 0.3	37.5 ± 0.3
NIA-86	77 ± 1.6	78 ± 1.6	24 ± 0.2	33 ± 0.1	2625 ± 14	3298 ± 6	27.2 ± 0.1	27.5 ± 0.1	35.1 ± 0.1	40.1 ± 0.5
NIA-M-30	40 ± 1.6	39 ± 0.6	34 ± 0.1	36 ± 0.1	4065 ± 6	4115 ± 9	28.2 ± 0.1	28.6 ± 0.2	37.5 ± 0.1	38.0 ± 0.6
NIA-HM-327	46 ± 1.6	44 ± 1.2	36 ± 0.2	38 ± 0.1	3788 ± 5	3850 ± 3	28.0 ± 0.3	28.5 ± 0.1	37.8 ± 0.1	39.5 ± 0.3
NIA-HM-329	73 ± 1.7	71 ± 0.6	26 ± 0.1	34 ± 0.1	2423 ± 13	3023 ± 13	28.3 ± 0.2	29.5 ± 0.1	33.5 ± 0.3	38.4 ± 0.4
NIA-HM-335	83 ± 0.6	79 ± 1.7	20 ± 10	36 ± 5.8	2230 ± 17	3121 ± 6	27.9 ± 0.1	28.6 ± 0.1	34.4 ± 0.2	36.0 ± 0.6
NIA-H-1	81 ± 0.6	79 ± 0.0	23 ± 0.1	33 ± 0.1	2869 ± 12	3454 ± 12	27.6 ± 0.1	28.6 ± 0.2	33.5 ± 0.0	37.5 ± 0.8
NIA-H-24	82 ± 0.6	80 ± 1.2	23 ± 0.1	34 ± 0.0	2551 ± 29	3209 ± 5	27.5 ± 0.0	27.6 ± 0.1	36.4 ± 0.0	38.4 ± 0.2
NIA-M31	50 ± 1.6	49 ± 0.6	36 ± 0.1	38 ± 0.1	3561 ± 1	3606 ± 3	28.0 ± 0.0	28.5 ± 0.1	37.5 ± 0.3	39.2 ± 0.1
NIA-M32	85 ± 0.6	83 ± 0.6	27 ± 0.1	30 ± 0.0	2320 ± 12	2855 ± 6	28.5 ± 0.1	20.4 ± 9.2	35.5 ± 0.7	38.3 ± 0.4
NIA-M33	69 ± 0.6	65 ± 0.6	27 ± 0.2	34 ± 0.1	2431 ± 18	2931 ± 18	28.9 ± 0.1	29.2 ± 0.1	36.6 ± 0.1	39.6 ± 0.6
NIA-M34	74 ± 0.6	71 ± 0.6	30 ± 0.1	36 ± 0.1	3054 ± 5	3356 ± 6	27.8 ± 0.1	28.5 ± 0.1	35.8 ± 0.3	39.4 ± 0.2
NIA-Perkh	51 ± 0.6	48 ± 1.2	32 ± 0.1	37 ± 0.1	3681 ± 6	3740 ± 3	28.0 ± 0.3	28.7 ± 0.2	36.6 ± 0.1	39.6 ± 0.1
NIA-H-13	68 ± 0.0	66 ± 0.6	21 ± 0.0	32 ± 0.0	2653 ± 30	3155 ± 6	27.0 ± 0.0	27.5 ± 0.1	37.5 ± 0.6	38.6 ± 0.1
NIA-HM-2	54 ± 0.6	53 ± 0.6	37 ± 0.1	40 ± 0.1	3898 ± 12	3936 ± 12	27.5 ± 0.1	28.1 ± 0.1	37.9 ± 0.6	38.7 ± 0.1
NIA-HM-48	51 ± 0.6	59 ± 0.6	36 ± 0.1	39 ± 0.1	3589 ± 6	3621 ± 12	28.7 ± 0.1	29.3 ± 0.1	36.2 ± 0.1	37.3 ± 0.6
NIA-M-2	84 ± 0.6	82 ± 1.2	22 ± 1.2	30 ± 0.7	2512 ± 7	3040 ± 23	27.8 ± 0.1	28.5 ± 0.1	33.3 ± 0.2	39.3 ± 0.8
NIA-HM-1	86 ± 0.6	83 ± 0.0	31 ± 2.7	35 ± 1.6	2456 ± 32	3470 ± 17	27.3 ± 0.1	27.5 ± 0.1	35.2 ± 0.1	38.6 ± 0.6
NIA-H-36	75 ± 0.6	77 ± 0.9	32 ± 1.0	39 ± 0.6	3132 ± 18	3441 ± 12	27.9 ± 0.1	28.5 ± 0.1	34.4 ± 0.1	37.4 ± 0.1
NIA-H-12	65 ± 0.6	63 ± 0.6	32 ± 0.1	33 ± 0.1	2923 ± 13	3255 ± 6	28.1 ± 0.1	28.4 ± 0.2	32.5 ± 0.6	39.2 ± 0.5
NIA-Bt-1	55 ± 0.6	50 ± 0.7	37 ± 0.1	36 ± 0.0	3768 ± 6	3820 ± 6	27.5 ± 0.1	28.2 ± 0.1	36.9 ± 0.6	38.5 ± 0.4
NIA-Bt-2	58 ± 0.6	55 ± 0.6	38 ± 0.2	40 ± 0.1	3526 ± 6	3690 ± 6	28.0 ± 0.0	28.3 ± 0.2	39.5 ± 0.3	40.3 ± 0.4
NIA-Bt-3	76 ± 0.6	76 ± 1.2	36 ± 0.1	39 ± 0.1	2959 ± 6	3415 ± 9	28.0 ± 0.1	28.5 ± 0.1	32.2 ± 0.6	37.4 ± 0.8
NIA-Bt-4	78 ± 0.6	80 ± 1.2	34 ± 0.1	37 ± 0.1	3046 ± 27	3570 ± 6	27.0 ± 0.0	27.9 ± 0.1	35.8 ± 0.6	37.9 ± 0.6
NIA-Bt-5	65 ± 0.6	65 ± 0.0	31 ± 0.1	38 ± 0.1	2427 ± 16	3254 ± 105	27.6 ± 0.1	28.0 ± 0.0	35.4 ± 0.2	37.5 ± 0.6
NIA-HM-320	79 ± 0.6	77 ± 0.0	30 ± 1.2	35 ± 0.7	2215 ± 9	3515 ± 9	28.2 ± 0.1	29.5 ± 0.1	33.4 ± 0.3	37.3 ± 0.2
NIA-HM-322	64 ± 0.0	60 ± 1.2	32 ± 0.2	35 ± 0.1	3022 ± 13	3356 ± 6	28.6 ± 0.1	29.0 ± 0.0	30.3 ± 0.2	32.6 ± 0.6
NIA-HM-323	78 ± 0.6	78 ± 0.0	31 ± 0.1	38 ± 0.1	2910 ± 6	3469 ± 6	28.0 ± 0.0	28.5 ± 0.1	32.5 ± 0.9	35.0 ± 0.6
NIA-HM-337	69 ± 0.6	67 ± 1.2	25 ± 0.1	35 ± 0.1	3041 ± 24	3445 ± 26	28.6 ± 0.1	29.0 ± 0.0	31.8 ± 0.6	34.9 ± 0.6
NIA-HM-338	65 ± 0.6	63 ± 1.7	22 ± 0.2	30 ± 0.1	3123 ± 13	3398 ± 6	28.2 ± 0.1	28.5 ± 0.2	36.2 ± 0.6	38.9 ± 0.1
NIA-HM-321	64 ± 0.6	64 ± 1.2	34 ± 0.1	37 ± 0.1	2236 ± 21	3236 ± 21	28.6 ± 0.2	29.0 ± 0.1	31.8 ± 0.6	34.1 ± 0.1
NIA-H-31	71 ± 0.6	70 ± 0.0	23 ± 0.1	30 ± 0.1	3240 ± 23	3523 ± 13	29.5 ± 0.1	30.0 ± 0.1	30.2 ± 0.1	32.5 ± 0.3
NIA-H-32	51 ± 0.6	50 ± 0.6	34 ± 0.1	37 ± 0.1	3439 ± 6	3539 ± 6	27.9 ± 0.1	28.5 ± 0.2	36.1 ± 0.6	38.1 ± 0.5
NIA-H-01	71 ± 0.6	70 ± 0.6	30 ± 0.1	35 ± 0.1	3144 ± 25	3463 ± 6	28.6 ± 0.1	29.3 ± 0.2	34.3 ± 0.1	35.5 ± 0.6
NIA-Okra-24	86 ± 0.6	84 ± 1.2	29 ± 0.2	39 ± 0.1	2229 ± 6	3433 ± 6	27.4 ± 0.1	28.5 ± 0.3	34.6 ± 0.6	35.3 ± 0.4
NIA-H-29	69 ± 0.6	67 ± 0.6	23 ± 0.8	33 ± 0.4	3022 ± 13	3687 ± 6	27.9 ± 0.1	28.1 ± 0.1	33.6 ± 0.6	38.5 ± 0.6
NIA-H-30	65 ± 0.6	68 ± 1.2	34 ± 0.6	36 ± 0.3	2918 ± 10	3332 ± 12	27.6 ± 0.1	28.3 ± 0.1	35.1 ± 0.6	37.2 ± 0.4
NIA-HM-311	74 ± 0.6	70 ± 0.6	32 ± 0.6	35 ± 0.3	2913 ± 8	3535 ± 20	27.0 ± 0.0	27.1 ± 0.1	33.3 ± 0.2	37.2 ± 0.1
NIA-H-67	73 ± 0.6	74 ± 0.6	24 ± 0.1	32 ± 0.1	2996 ± 6	3296 ± 2	27.5 ± 0.1	27.8 ± 0.1	33.5 ± 0.3	35.3 ± 0.4
CRIS-121	83 ± 1.2	81 ± 0.6	34 ± 0.6	36 ± 0.3	2763 ± 6	3301 ± 2	28.0 ± 0.3	28.1 ± 0.1	33.3 ± 0.4	36.5 ± 0.6
CRIS-9	65 ± 0.6	68 ± 0.0	33 ± 0.4	38 ± 0.3	3002 ± 1	3532 ± 18	28.2 ± 0.1	28.5 ± 0.1	35.6 ± 0.3	39.6 ± 0.6
Sindh-1	67 ± 0.6	69 ± 0.6	30 ± 0.1	36 ± 0.0	2650 ± 29	3201 ± 3	27.2 ± 0.1	27.5 ± 0.1	34.8 ± 0.6	38.1 ± 0.5
Hari Dost	75 ± 0.0	73 ± 0.6	22 ± 0.6	32 ± 0.3	3108 ± 5	3532 ± 18	27.8 ± 0.1	28.0 ± 0.0	33.6 ± 0.6	38.2 ± 0.1
Sohni	68 ± 0.6	64 ± 0.6	32 ± 0.2	34 ± 0.1	3021 ± 12	3444 ± 2	27.9 ± 0.1	28.0 ± 0.0	36.4 ± 0.7	39.3 ± 0.4
NIA-Ufaq	63 ± 1.7	62 ± 1.2	34 ± 0.5	36 ± 0.3	3420 ± 12	3520 ± 12	28.0 ± 0.0	28.4 ± 0.1	35.2 ± 0.1	38.5 ± 0.6
CRIS-342	62 ± 1.2	62 ± 1.2	31 ± 0.7	32 ± 0.4	3331 ± 18	3356 ± 6	27.5 ± 0.3	28.1 ± 0.1	35.5 ± 0.6	38.3 ± 0.2
Chandi-95	65 ± 0.6	62 ± 1.2	33 ± 0.6	37 ± 0.3	3118 ± 10	3630 ± 17	29.6 ± 0.1	31.2 ± 0.1	33.4 ± 0.2	36.4 ± 0.2
Sadori	51 ± 0.0	50 ± 1.2	36 ± 0.2	37 ± 0.1	3542 ± 24	3570 ± 17	28.3 ± 0.1	28.8 ± 0.1	37.6 ± 0.6	38.5 ± 0.6
Shahbaz	72 ± 0.6	71 ± 0.6	28 ± 0.1	32 ± 0.0	2354 ± 31	3099 ± 1	28.0 ± 0.0	28.2 ± 0.1	34.5 ± 0.3	38.2 ± 0.1
IR-3701	65 ± 0.6	64 ± 0.9	32 ± 0.1	36 ± 0.1	3332 ± 6	3390 ± 6	27.5 ± 0.2	28.0 ± 0.0	34.2 ± 0.5	37.1 ± 0.5
NIAB-78	70 ± 1.2	65 ± 0.6	27 ± 0.1	33 ± 0.1	3053 ± 6	3420 ± 12	27.2 ± 0.1	27.6 ± 0.1	30.7 ± 0.6	34.8 ± 0.1
NIAB-111	64 ± 1.2	62 ± 1.2	34 ± 0.2	36 ± 0.1	3489 ± 6	3526 ± 6	28.0 ± 0.0	28.3 ± 0.1	35.6 ± 0.6	36.4 ± 0.6
CIM-469	80 ± 0.6	77 ± 0.6	29 ± 0.6	29 ± 0.3	3122 ± 6	3290 ± 3	27.6 ± 0.1	28.0 ± 0.0	34.4 ± 0.6	35.2 ± 0.1
CRIS-134	50 ± 1.2	47 ± 0.6	32 ± 0.1	34 ± 0.1	3556 ± 6	3590 ± 6	27.8 ± 0.1	28.0 ± 0.1	36.7 ± 0.5	37.6 ± 0.3

*RCI%, relative cell injury percentage, GOT%, ginning out turn percentage, SD 1, sowing date 1 (15 March), SD 2, sowing date 2 (15 May).*

The SCY of 58 genotypes are presented in Table 1. SCY in studied genotypes ranged from 2,215 to 4,115 kg/ha under heat stress and non-heat stress conditions. The selected seventeen heat-tolerant genotypes performed well in both regimes. NIA-M-30 had the highest SCY (4,115 kg/ha) followed by NIA-HM-2 (3,936 kg/ha) and NIA-80 (3,912 kg/ha). NIA-HM-320 produced lowest SCY (2,215 kg/ha) under heat stress conditions. The HSI based on SCY varied in different genotypes (Table 2). In the present study, HSI values ranged between 0.32 and 10.85. Superior heat-tolerant genotypes gave the least values of HSI < 1 and high yield under heat stress conditions. Whereas, other genotypes, which were found as heat susceptible, had a higher HSI.

**TABLE 2:** Heat susceptible index (HSI) of 58 cotton genotypes for yield and fiber traits.

Genotypes	NBP	SCY	SL	GOT%	Genotypes	NBP	SCY	SL	GOT%
NIA-80	0.59	0.31	0.56	1.19	NIA-HM-320	4.41	5.10	1.32	1.52
NIA-81	0.56	0.50	0.52	0.68	NIA-HM-322	3.05	2.97	1.54	2.06
NIA-83	0.32	0.20	0.32	0.84	NIA-HM-323	5.08	4.81	1.15	1.30
NIA-84	0.46	0.26	0.31	1.45	NIA-HM-337	8.48	3.50	1.03	1.80
NIA-85	2.72	2.39	1.07	1.56	NIA-HM-338	7.81	2.42	1.36	1.28
NIA-86	8.00	3.09	1.17	1.49	NIA-HM-321	2.41	4.53	1.44	1.56
NIA-M-30	0.34	0.36	0.42	0.38	NIA-H-31	6.78	2.40	1.19	2.09
NIA-HM-327	0.95	0.48	0.52	1.26	NIA-H-32	0.33	0.84	0.63	0.90
NIA-HM-329	7.33	3.29	1.21	1.44	NIA-H-01	4.31	2.75	1.32	1.07
NIA-HM-335	3.44	2.18	1.15	1.36	NIA-Okra-24	7.48	4.80	1.15	0.63
NIA-H-1	2.70	2.70	1.04	1.55	NIA-H-29	8.77	5.38	1.17	2.31
NIA-H-24	10.10	4.54	1.67	1.56	NIA-H-30	2.14	3.71	1.16	1.71
NIA-M31	0.86	0.37	0.52	1.29	NIA-HM-311	2.30	5.25	1.17	1.44
NIA-M32	3.48	4.71	1.03	2.14	NIA-H-67	7.97	2.72	1.35	1.58
NIA-M33	6.02	2.27	1.12	1.53	CRIS-121	2.23	4.87	1.06	1.74
NIA-M34	4.81	2.69	1.26	1.43	CRIS-9	4.05	4.48	1.05	1.48
NIA-Perkh	0.64	0.50	0.69	0.73	Sindh-1	4.61	5.14	1.27	1.02
NIA-H-13	10.85	4.75	1.57	0.85	Hari Dost	9.95	3.58	0.53	1.25
NIA-HM-2	0.60	0.29	0.64	0.59	Sohni	1.66	3.67	1.16	1.44
NIA-HM-48	0.54	0.26	0.61	0.86	NIA-Ufaq	1.80	0.85	0.42	1.76
NIA-M-2	7.66	4.35	0.73	4.56	CRIS-342	0.62	0.28	0.69	0.68
NIA-HM-1	3.46	5.17	1.26	2.59	Chandi-95	2.86	4.21	1.53	1.64
NIA-H-36	5.19	2.68	1.05	2.39	Sadori	0.64	0.23	0.52	0.70
NIA-H-12	1.44	3.04	1.14	5.08	Shahbaz	3.80	5.61	1.27	2.17
NIA-Bt-1	0.32	0.41	0.74	1.24	IR-3701	0.67	0.51	0.53	2.35
NIA-Bt-2	0.61	0.84	0.53	0.59	NIAB-78	6.02	3.20	1.15	1.88
NIA-Bt-3	2.00	3.99	1.26	1.75	NIAB-111	0.82	0.31	0.73	0.62
NIA-Bt-4	2.26	4.38	1.27	1.62	CIM-469	1.61	1.44	1.05	0.67
NIA-Bt-5	5.56	3.54	1.26	1.68	CRIS-134	0.64	0.32	0.43	0.75

*NBP, no. of bolls/plant, SCY, seed cotton yield, SL, staple length (mm), GOT%, ginning out turn.*

For the determination of heat tolerance, the data for bolls/plant in cotton genotypes were also considered. The mean values of number of bolls/plant showed that both heat stress and non-stress conditions had differently affected the boll retention capacity of fifty-eight genotypes. The heat-tolerant genotypes produced maximum bolls/plant in both heat stress and non-stress regimes. While other genotypes produced less number of bolls as a result of high temperature (Table 1). Superior heat-tolerant genotypes produced maximum bolls/plant under both sowing condition indicating that these genotypes could be less affected by heat stress (Table 2).

Table 1 represents the mean values of GOT% of studied genotypes, which ranged from 30 to 40.56% as a result of early and normal sowing dates. Less effects of high temperature stress were recorded in terms of GOT% in NIA-M-30, NIA-HM-48, NIA-H-32, NIA-Bt1, NIA-Perkh, CRIS-342, NIAB-111, CRIS-134, and Sadori. Non-significant differences in mean values were observed for the staple length (in mm). However, the staple length for all genotypes ranged from 27 to 30 mm. A decrease in fiber length can be attributed to high temperature. But in the present study, eighteen genotypes showed less reduction in the staple length and gave the lowest value of HSI < 1 (Table 2).

### Scanning Electron Microscopy Study of Stomata and Trichome Size of Heat-Tolerant Cotton Genotypes

**FIGURE 1:**
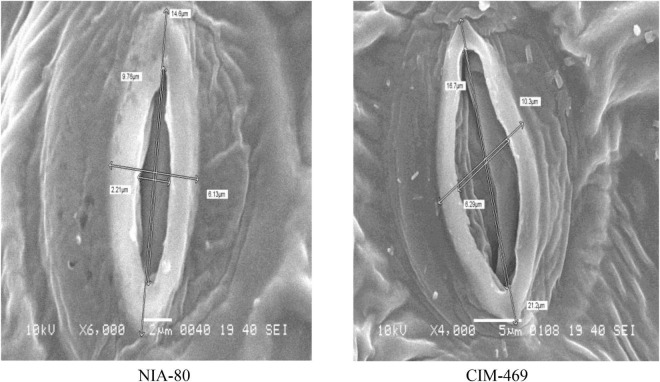
Difference in stomata size of heat-tolerant (NIA-80) and heat-susceptible (CIM-469) cotton genotypes revealed through scanning electron microscopy (SEM).

**FIGURE 2:**
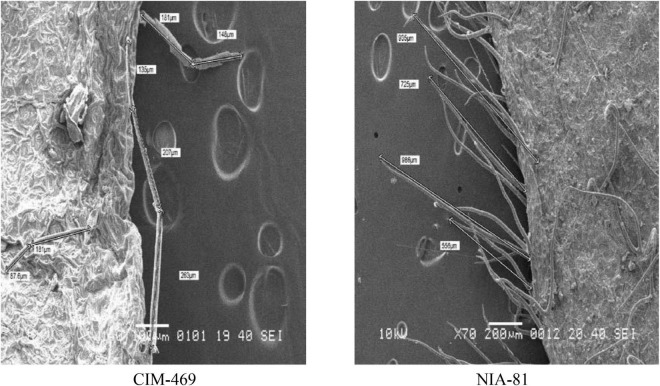
Difference in trichome size of heat-susceptible (CIM-469) and heat-tolerant (NIA-81) cotton genotypes revealed through SEM.

Highly significant and significant differences among genotypes were observed for the stomata and trichome size, respectively (Supplementary Table 3). The stomata and trichome size of different genotypes examined with SEM are presented in Table 3, and the difference in stomata and trichome size of heat-tolerant and heat-susceptible genotypes is shown in Figures 1, 2. The minimum value of the stomata size on the surface of cotton leaf was recorded as 21.57 μm^2^, and the maximum stomata size was recorded as 105 μm^2^ with ± 23.3 standard deviation. The large size of stomata was observed in genotype CIM-469 (105.0 μm^2^) followed by genotype NIAB-111 (87.0 μm^2^). Whereas, two genotypes, namely, NIA-80 (21.6 μm^2^) and NIA-M30 (22.6 μm^2^), gave small size stomata. The results of the electron micrograph obtained from the leaf epidermis tissues showed that all genotypes were non-glandular single-cell trichome. All the selected genotypes showed a high density of trichomes and large trichome size as compared with susceptible check. Among them, the highest average size and density of trichome were recorded (782.7 μm) in genotype NIA-81 followed by Sadori (468 μm) and NIA-80 (465 μm). Whereas, the average lowest density and trichome size (177 μm) were observed in the susceptible check (i.e., CIM-469). The standard deviation value for the minimum trichome length of all genotypes was ± 156.40, and maximum trichomes lengths were ± 144.12. The average standard deviation was observed to be ± 136.31.

**TABLE 3:** Mean stomata and trichome size of selected 18 cotton genotypes.

Genotypes	Open stomata			Trichome size (μm)		
	Length (μm)	Width (μm)	Size (μm^2^)	Minimum value	Maximum value	Average
NIA-80	9.76	2.21	21.6 i	420	519	465.0 bc
NIA-81	15.2	4.77	72.5 e	725	823	782.7 a
NIA-83	14.8	4.96	73.4 c	234	790	419.3 bc
NIA-84	11.8	2.78	32.8 g	216	490	328.7 b-e
NIA-H32	11.9	3.64	44.3 f	89.6	510	348.2 b-e
NIA-M-30	7.81	2.89	22.6 i	147	372	257.0 c-e
NIA-HM-327	16.7	6.29	39.2 f	109	322	184.7 de
NIA-M31	14.3	2.88	41.2 f	311	685	446.0 bc
NIA-Perkh	9.95	2.68	26.7 h	160	634	438.0 bc
NIA-HM-2-1	11.1	3.18	35.3 g	73.5	584	256.8 c-e
NIA-HM-48	16.8	3.44	57.8 d	222	490	374.3 b-e
NIA-Bt-1	12.9	3.52	45.4 e	131	545	402.0 bc
NIA-Bt-2	11.8	5.01	59.1 d	176	471	317.7 b-e
NIAB-111	15.4	5.65	87.0 b	130	503	318.0 b-e
Sadori	12.7	3.65	46.4 e	317	557	468.0 b
CRIS-342	12.01	4.03	48.4 h	164	410	369.0 b
CIM-469	16.7	6.29	105.0 a	87	263	177.0 e
CRIS-134	13.2	4.47	56.0 d	143	565	388.0 b-d
Minimum value	7.81	2.21	21.57	73.5	263	177
Maximum value	16.8	6.29	105.04	725	823	782.7
Standard deviation	±2.63	±1.11	±23.23	±156.40	±144.12	±136.31

### Association of Relative Cell Injury Percentage, Stomata, and Trichome Size With Seed Cotton Yield and Ginning Out Turn Percentage

**FIGURE 3:**
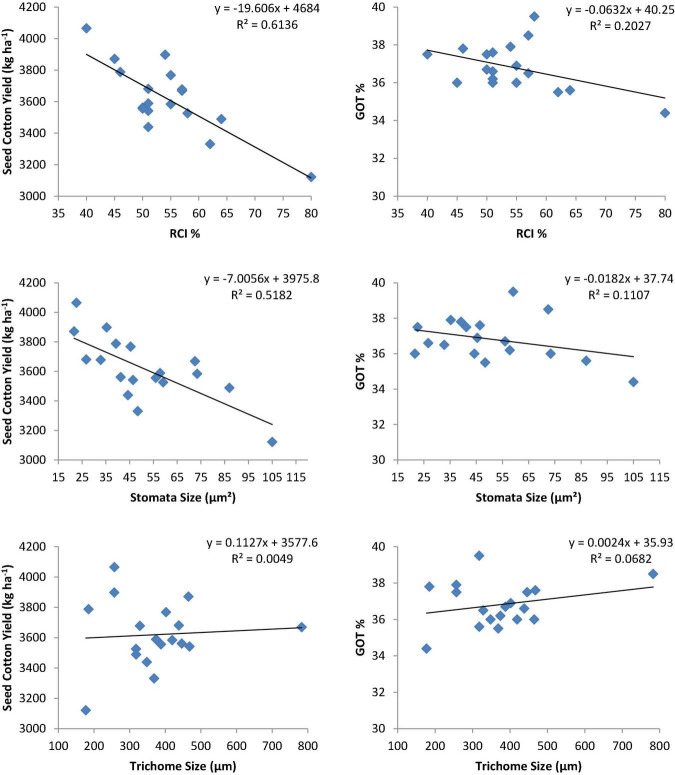
Relationship of physiological traits with seed cotton yield (kg ha^– 1^) and ginning out turn percentage (GOT%) of selected heat-tolerant cotton genotypes.

Coefficient of determination (R^2^) depicted that the variation in SCY was due to the effects of RCI% and stomata size (Figure 3). This shows that a higher SCY among genotypes was significantly associated with a lower RCI% and stomata size. Therefore, the regression analysis illustrated that the RCI% and stomata size had a strong negative effect on SCY, with coefficients of determination (R^2^) 0.61 and 0.51, respectively. The trichome size exhibited positive but non-significant relationships with SCY. Relationships between RCI%, stomata size, and trichome size with GOT% were observed to be weak or non-significant.

### G × E Interaction Study of Selected Heat Tolerant Cotton Genotypes

Mean squares showed highly significant (*P* < 0.01) differences for mean SCY among genotypes, environments, and the G × E interaction (Supplementary Table 4). Highly significant differences among locations indicated the presence of the G × E interaction in this area. The stability analysis showed the presence of significant G × E interactions for SCY in this region. As per results, all selected genotypes showed higher yield and stability as compare to susceptible check. Among the genotypes, NIA-M30, NIA-80, and NIA-83 produced a high average yield (3.47, 3.47, and 3.31 tons/ha) and wide adaptability with unit regression (bi∼1) and non-significant deviation from the regression line (S^2^d∼0). These genotypes are high yielding and stable (Table 4) and may be tested over all environments to attain an additional stable performance. Moreover, three genotypes, namely, NIA-M31, NIA-HM48, and NIA-Bt2, were considered suitable for the specific environment by way of expected performance as they possess a high seed yield (ton/ha) with increased regression average (bi > 1). Five other genotypes viz., NIA-Perkh, NIA-Bt2, Sadori, CRIS-134, and CIM-469 were found suitable to poor environments as they showed good performance in the seed yield with regression (bi < 1) and above average non-significant deviation from the regression (Table 4).

**TABLE 4:** Seed cotton yield (tons/ha) and stability parameters for SCY of 18 selected genotypes tested over different locations during 2017–2018.

Genotypes	L1	L2	L3	L4	L5	Overall genotypic mean yield	b ± SE (b)	S^2^d
NIA-80	3.668	3.570	3.240	3.054	3.055	3.317 b	1.186 ± 0.123	0.003
NIA-81	3.495	3.364	3.133	2.858	2.828	3.135 ef	1.240 ± 0.055	0.000
NIA-83	3.593	3.404	3.303	3.150	3.033	3.297 b	0.900 ± 0.091	0.002
NIA-84	3.343	3.186	2.779	2.447	2.481	2.847 h	1.684 ± 0.138	0.004
NIA-H32	2.943	3.559	2.915	2.547	2.788	2.950 g	0.964 ± 0.714	0.116
NIA-M-30	3.867	3.471	3.554	3.249	3.216	3.471 a	1.021 ± 0.242	0.013
NIA-HM-327	3.694	2.993	3.330	3.045	2.952	3.203 d	0.941 ± 0.523	0.062
NIA-M31	3.503	2.879	2.901	2.718	2.632	2.926 g	1.269 ± 0.379	0.033
NIA-Perkh	3.381	3.585	3.309	3.143	3.176	3.319 b	0.587 ± 0.263	0.016
NIA-HM-2-1	3.579	2.927	3.228	3.082	3.086	3.180 d-f	0.563 ± 0.501	0.057
NIA-HM-48	3.389	3.343	2.777	2.583	2.671	2.952 g	1.522 ± 0.300	0.020
NIA-Bt-1	3.570	3.466	3.131	2.957	2.814	3.187 de	1.335 ± 0.139	0.004
NIA-Bt-2	3.567	3.420	3.272	3.126	3.035	3.284 bc	0.897 ± 0.056	0.000
NIAB-111	3.145	3.153	2.954	2.855	2.762	2.974 g	0.697 ± 0.120	0.003
Sadori	3.476	3.360	3.267	3.069	2.977	3.230 cd	0.848 ± 0.083	0.002
CRIS-342	3.388	3.278	3.128	2.945	2.857	3.119 f	0.922 ± 0.063	0.000
CIM-469	2.927	2.774	2.660	2.501	2.439	2.660 i	0.831 ± 0.039	0.000
CRIS-134	2.962	2.971	2.856	2.684	2.664	2.827 h	0.592 ± 0.099	0.002
Overall site mean yield	3.416a	3.261b	3.096c	2.889d	2.859e			

*L1, location 1 (Tando Jam), L2, location 2 (Matiari), L3, location 3 (Shaheed Benazirabad), L4, location 4 (Khairpur), L5, location 5 (Dadu), b, variance due to regression coefficient, SE, standard error, S^2^d, deviation from regression coefficient.*

### Genetic Improvement Through Hybridization for Heat Tolerance and Genetic Analysis of Relative Cell Injury and Seed Cotton Yield in F_1_ and F_2_ Generations

This research work was carried out to find out combining ability variance effects of parents and their F_1_ hybrids of cotton and their efficient use in the improvement of breeding program. The ANOVA (Supplementary Table 5) reveals that the mean squares for the interaction of temperature regimes were highly significant (*P* < 0.01) for parents and their crosses. A significant variation was identified for RCI% in parents as well as their respective crosses under field regimes. Parents vs. crosses mean square, an indicator of average heterosis, was also significant under field regimes (*P* < 0.01), indicating that the mean RCI% among crosses deviated substantially from the mean of their parental cultivars. ANOVA of combining ability for RCI% and SCY (g/plant) was computed separately using two temperature regimes (March and May) indicated a significant variation for RCI% due to lines and testers (*P* < 0.01) under both regimes in the field.

### Mean Performance of Parents and Their Hybrids for Relative Cell Injury and Seed Cotton Yield in F_1_

In the presents study, lower RCI was interpreted as higher CMT due to heat sensitivity. The variation among parents as well as hybrids was significant (*P* < 0.01) for RCI under field and glasshouse regimes. The mean performance of parents and their hybrids for RCI% and SCY (g/plant) is presented in Table 5. Among parents, RCI ranged between 43 and 78% in the glasshouse and between 42 and 75% in the field, whereas tester NIA-Perkh, CRIS-342, and CRIS-134 showed minimum RCI in glasshouse (i.e., heat stress) and field (i.e., non-stress) regimes, respectively; however, line NIA-148 showed maximum RCI in both glasshouse and field temperature regimes. Among F_1_ hybrids, RCI ranged between 42 and 70% in the glasshouse and 42 and 67% in the field regime. NIA-Perkh, CRIS-342, and CRIS-134 among parents had lowest, while NIA-148, Haridost, and Sohni had the highest relative cell injury percent. The crosses NIA-148 × NIA-Perkh, Sohni × CRIS-134, and NIA-148 × CRIS-342 had lowest RCI%, which showed stability in the cell membrane among hybrids was below average in both glasshouse and field regimes. Hybrid NIA-148 × CRIS-134 had maximum RCI%.

**TABLE 5:** Mean performance of parents and their hybrids for RCI% and SCY (g/plant) in F_1_ and F_2_ generations.

Parents and hybrids	F_1_ generation				F_2_ generation			
	RCI%		SCY (g/plant)		RCI%		SCY (g/plant)	
	Glasshouse	Field	Glasshouse	Field	Glasshouse	Field	Glasshouse	Field
CRIS-342	51	49.3	118.4	125.4	50	49.3	116.73	118
NIA-Perkh	43	42.3	113.4	118.9	40	38.9	121.46	115
CRIS-134	52	51.7	108.5	111.9	48	50	109.52	118
Haridost	71	71	72.6	95.7	75.3	74.3	72.81	121
Sohni	70	71.7	76.7	108.2	76	75.1	72.92	97
NIA-148	78	78.4	81.2	118.8	80	82.3	62.88	116
Haridost × CRIS-342	45.1	45.2	130.3	140.9	38.3	37.5	145	145
Haridost × NIA-Perkh	50	48.1	102.9	138.5	44.4	42.6	134.79	140
Haridost × CRIS-134	55	56.7	109.3	138	51	51	128.12	142
Sohni × CRIS-342	51	49.2	129.1	135.9	47.6	46.6	166.63	167
Sohni × NIA-Perkh	44.1	42.6	135.4	146.1	50.8	48.2	113.57	133
Sohni × CRIS-134	51	51.4	96.5	122.9	60	58.7	132	132
NIA-148 × CRIS-342	43	43.3	133.3	144.5	42.6	41.1	144.99	148
NIA-148 × NIA-Perkh	42.1	42.4	129.5	140.6	51.1	50.1	139.63	142
NIA-148 × CRIS-134	70	67.2	89.6	129.2	72.9	72.7	81.99	124

**FIGURE 4:**
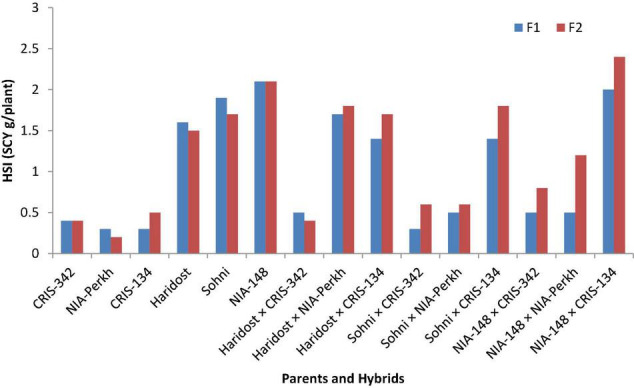
Heat susceptibility index (HSI) for SCY (g/plant) among parents and their progenies in F_1_ and F_2_ generations.

The results regarding stocktickerSCY (g/plant) are presented in Table 5, which showed that the testers stocktickerCRIS-324, NIA-Perkh, and stocktickerCRIS-134 produced the highest SCY, whereas, three lines gave the lowest yield under both temperature regimes. Among the crosses, HariDost × stocktickerCRIS-342, NIA-148 × NIA-Perkh, NIA-148 × stocktickerCRIS-342, Sohni × stocktickerCRIS-342, and Sohni × stocktickerCRIS-134 showed better performance under non-heat stress and heat stress regimes. The HSI for parents and F_1_ hybrids was ranged from 0.32 to 2.0%. The parents and hybrids with a higher stocktickerSCY performance have also shown lowest values of HSI. The superior genotypes for heat tolerance gave the least values of HSI (HSI < 1) and high yield under heat stress conditions (Figure 4).

### Combining Ability Variance and Effects

**FIGURE 5:**
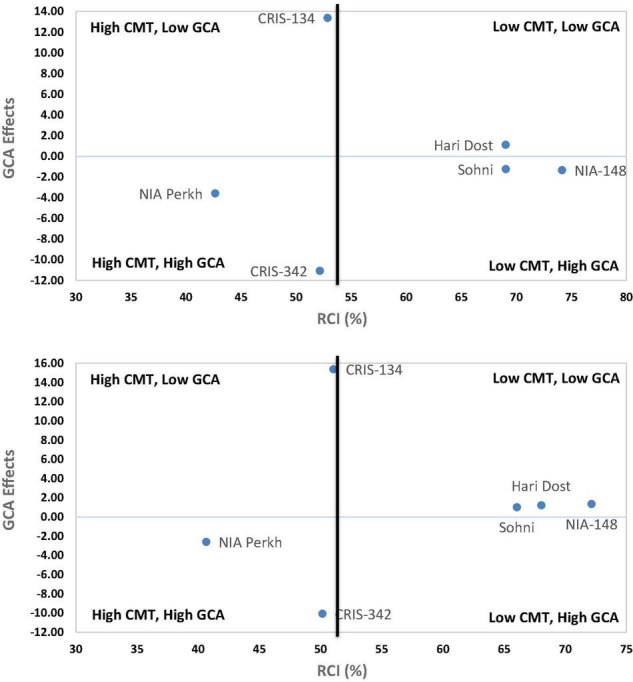
Scatter diagram between RCI% and general combining ability (GCA) effects in upland cotton genotypes (parents) in the glasshouse (up) and field (down) data based on F_1_ generation.

**FIGURE 6:**
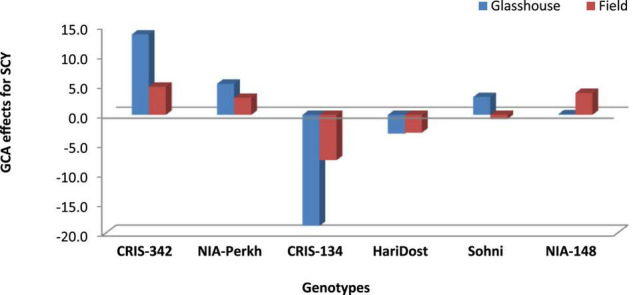
Estimates of GCA effects for SCY (g/plant) in six parents data based on F_1_ generation.

**FIGURE 7:**
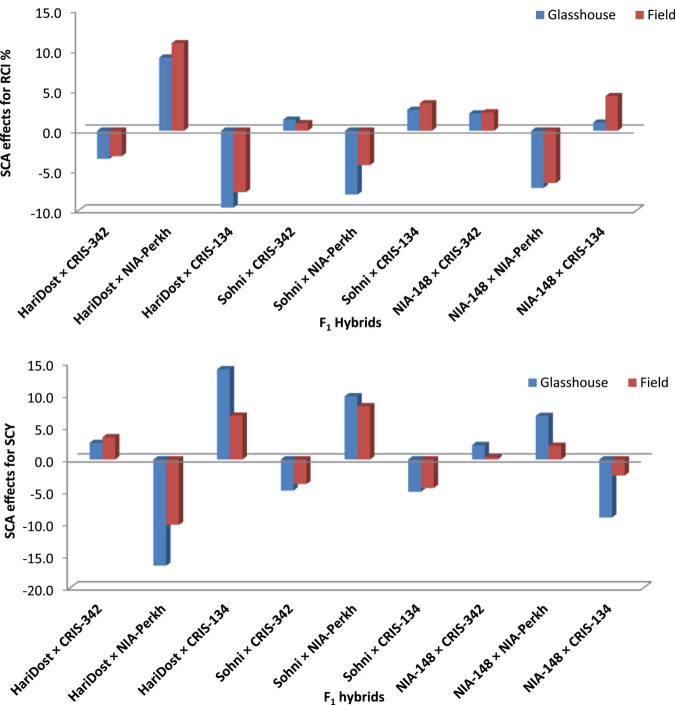
Estimates of SCA effects for RCI% and SCY (g/plant) in F_1_ generation of cotton genotypes in heat-stress (glasshouse) and non-heat-stress (field) conditions.

The effects of general combining ability associated with parental varieties were computed for RCI% under glasshouse and field conditions. The scatter diagram (Figure 5) revealed that CRIS-342 and NIA-Perkh could be categorized in high CMT and high GCA group under both temperature regimes, therefore, could be used as a suitable parent in breeding programs aimed to improve CMT in cotton. Cultivars CRIS-134, CRIS-342, NIA-Perkh, and Sohni were recognized in low CMT and low GCA group under both regimes. The variety CRIS-134, NIA-148, and Haridost showed positive GCA effects. The varieties NIA-148 and Haridost were recognized in low CMT and low GCA group under both temperature regimes, indicated that these cultivars will not be suitable as donor parents for CMT studies. For SCY, the parents NIA-Perkh, CRIS-342, and Sohni displayed the positive GCA (Figure 6). Three tolerant (based on CMT) genotypes expressed differential patterns of GCA effects and also show differential response (i.e., SCA effects) in different cross combinations. The combinations NIA-148 × CRIS-342, Haridost × CRIS-342, Sohni × CRIS-134, and NIA-148 × NIA-Perkh had a negative RCI% value, which proved to be the best indicator showing tolerance to heat stress in hybrids (based on CMT) in both regimes. Three hybrids Sohni × CRIS-342, Sohni × NIA-Perkh, and NIA-148 × CRIS-134 showed a positive RCI%, indicating susceptibility to heat stress. Sohni × NIA-Perkh and NIA-148 × CRIS-342 crosses proved as the best combiners for SCY (Figure 7).

### Heterotic Effects in F_1_ Generation

The heterosis values for RCI% and SCY (g/plant) expressed as the percentage increase or decrease over mid-parent (i.e., relative heterosis or mid parent heterosis) and over better parent (i.e., hetrobeltiosis) are presented in Table 6. Results revealed that there was a general tendency of increase in the cell membrane stability of hybrids over mid- and better parents, respectively. Negative heterotic effects for RCI (%) indicated less damage to cellular membranes and vice versa. In the present study, maximum negative mid-parent and better parent heterosis were observed in HariDost × CRIS-342, Sohni × CRIS-342, NIA-148 × CRIS-342, and NIA-148 × NIA-Perkh, while the maximum positive heterotic effects were produced by hybrids NIA-148 × CRIS-134 and HariDost × NIA-Perkh under both temperature regimes. The results of heterosis for SCY (g/plant) have shown maximum significant positive heterosis and heterobeltiosis under both heat stress and non-heat stress regimes in the crosses HariDost × CRIS-342, Sohni × CRIS-342, Sohni × NIA-Perkh, NIA-148 × CRIS-342, and NIA-148 × NIA-Perkh. However, maximum negative heterotic effects were observed in cross NIA-148 × CRIS-134 in heat stress conditions (i.e., glasshouse).

**TABLE 6:** Heterotic effects for RCI% and SCY (g/plant) in F_1_ generation.

Hybrids	RCI%				SCY (g/plant)			
	Glasshouse		Field		Glasshouse		Field	
	MPH	BPH	MPH	BPH	MPH	BPH	MPH	BPH
**F_1_ Generation**								
Haridost × CRIS-342	−25	−7.8	−23	−8.4	36	10.1	27	12.3
Haridost × NIA-Perkh	−15	14.6	−13	13.7	10	−9.3	29	16.5
Haridost × CRIS-134	−8	9	−5	9.7	21	0.7	33	23.4
Sohni × CRIS-342	−18	0.5	−14	−0.2	32	9.1	16	8.4
Sohni × NIA-Perkh	−10	22.3	−4	21.3	42	19.4	29	22.8
Sohni × CRIS-134	−31	−18.1	−27	−17.5	4	−11.1	12	9.8
NIA-148 × CRIS-342	−32	−11.6	−27	−12.2	34	12.6	18	15.2
NIA-148 × NIA-Perkh	−29	0.9	−25	0.1	33	14.2	18	18.2
NIA-148 × CRIS-134	3	29.2	10	30.0	−6	−17.4	12	15.5

*MPH, mid-parent heterosis; BPH, better parent heterosis.*

### Performance of Parents and Their Hybrids for Relative Cell Injury and Seed Cotton Yield in F_2_ Generation

The results for RCI and SCY in parents and F_2_ progenies are presented in Table 5. Among parents, RCI ranged between 40 and 80% in glasshouse and between 38 and 82% in field conditions. Tester NIA-Perkh, CRIS-342, and CRIS-134 showed minimum RCI in both regimes, whereas line NIA-148 showed maximum RCI in both glasshouse and field conditions. Among F_2_progenies, RCI ranged between 38 and 72% in the glasshouse and 37 to 72% in field conditions. NIA-Perkh, CRIS-342 and CRIS-134 among parents had lowest, while NIA-148, Haridost, and Sohni had the highest relative cell injury percent. The crosses NIA-148 × NIA-Perkh, HariDost × CRIS-342, Sohni × CRIS-342, and NIA-148 × CRIS-342 had a lowest relative cell injury level percentage, which showed the stability in cell membrane among hybrids was below average RCI in both heat stress (i.e., glasshouse) and non-stress (i.e., field) regimes. For SCY, the results showed that the testers CRIS-324, NIA-Perkh, and CRIS-134 produced the highest SCY, whereas, three lines gave the lowest SCY under both temperature regimes. Among the crosses, HariDost × CRIS-342, HariDost × NIA-Perkh, Sohni × CRIS-342, Sohni × NIA-Perkh, and NIA-148 × NIA-Perkh showed better performance under both temperature regimes.

Values of HSI in parents and F_2_ progenies (Figure 4) were ranged between 0.2 and 2.3%. Genotypes NIA-Perkh, CRIS-342, and CRIS-134 gave the lowest value for HSI. Among the nine crosses, five crosses HariDost × CRIS-342, Sohni × NIA-CRIS-342, Sohni × NIA-Perkh, NIA-148 × NIA-Perkh, and NIA-148 × CRIS-342 gave the lowest value for HSI. The superior genotypes for heat tolerance gave the least values (HSI < 1) and high yield under heat stress conditions.

### Genetic Parameters of Relative Cell Injury and Seed Cotton Yield in F_2_ Generation

As far as heritability and genetic advance for RCI% are concerned, crosses HariDost × CRIS-342, Sohni × CRI-342, Sohni × NIA-Perkh, and NIA-148 × NIA-Perkh displayed a high heritability and moderate genetic advance under both temperature regimes, while other crosses displayed a low heritability and low genetic advance. Lowest heritability and genetic advance were recorded in cross NIA-148 × CRIS-134 (Table 7).

**TABLE 7:** Genetic studies of RCI% and SCY (g/plant) in F_2_ progenies.

Traits	F_2_ Progenies	Genetic variance (σ^2^g)		Heritability broad sense (h^2^%)		Genetic advance (GA)	
		Glasshouse	Field	Glasshouse	Field	Glasshouse	Field
RCI%	Haridost × CRIS-342	5.10	22.0	82.95	87.2	20.23	21.0
	Haridost × NIA-Perkh	38.58	64.9	55.43	65.3	12.17	16.0
	Haridost × CRIS-134	61.42	51.2	51.28	65.8	15.42	11.3
	Sohni × CRIS-342	11.0	6.4	87.66	89.6	16.40	19.5
	Sohni × NIA-Perkh	53.42	17.8	92.10	88.1	14.45	17.1
	Sohni × CRIS-134	26.41	76.3	50.58	41.4	9.50	7.2
	NIA-148 × CRIS-342	3.76	26.6	60.05	45.4	3.09	7.2
	NIA-148 × NIA-Perkh	11.01	5.5	90.64	84.1	6.51	1.8
	NIA-148 × CRIS-134	145.04	86.4	43.94	53.3	11.34	10.4
SCY (g/plant)	Haridost × CRIS-342	783.45	237.8	88.67	84.4	54.29	41.2
	Haridost × NIA-Perkh	330.52	539.7	44.48	59.4	12.32	19.9
	Haridost × CRIS-134	1141.1	621.6	42.22	58.6	6.83	4.0
	Sohni × CRIS-342	1571.2	364.2	95.23	75.4	59.69	54.1
	Sohni × NIA-Perkh	1439.7	539.3	94.00	90.1	55.79	65.4
	Sohni × CRIS-134	521.90	840.6	47.46	42.6	4.01	4.3
	NIA-148 × CRIS-342	1116.6	1345.9	93.26	87.7	66.48	70.8
	NIA-148 × NIA-Perkh	519.96	564.0	85.37	81.3	43.40	44.1
	NIA-148 × CRIS-134	173.33	376.6	51.43	60.4	5.50	3.1

For SCY, out of nine crosses, five crosses showed a high heritability along with high genetic advance. Sohni × CRIS-342, Sohni × NIA-Perkh, NIA-148 × CRIS-342, and HariDost × CRIS-342 displayed the highest heritability coupled with genetic advance under both temperature regimes, which is indicative of the additive type of gene action. However, NIA-148 × CRIS-134 and Sohni × CRIS-134 displayed the low heritability estimates coupled with low genetic advance (Table 7).

## Discussion

In the present study, the temperature at peak flowering in early sowing (March) was high (44.2/32.2°C) as compared with normal sowing (39.8/28.4°C) during the month of May. Both temperature regimes in the field were able to expose the studied genotypes to differential responses, resulting in significant sowing the date × genotypes interaction. Previously, Rahman et al. (2004) reported a highly significant genotype × temperature interaction in field. The present study reports seventeen heat-tolerant genotypes, identified on the basis of low RCI% (40–50) and HSI (<1), higher boll retention, and SCY. Differential responses of genotypes to heat in terms of RCI were consistent under heat stress and non-stress regimes. RCI is an indicator of cellular heat tolerance, i.e., high RCI (>70%) means low CMT and vice versa (Khan et al., 2008). Heat-tolerant genotypes produced a high SCY with high GOT% and staple length, while susceptible genotypes were low yielding with inferior fiber traits. Our results are in confirmation with the reports of Azhar et al. (2009) and Karademir et al. (2012). HSI was also used as an extent of relative decrease in yield influenced by non-favorable vs. favorable environments. Results indicated the values of HSI < 1 for heat-tolerant genotypes. Previously, Hafeez-ur-Rahman (2006) measures the heat tolerance in cotton at the whole-plant level using the heat-tolerant index. The SEM study of heat-tolerant genotypes along with susceptible check revealed a wide range of stomata and trichome size present on leaves of these genotypes. Galdon-Armero et al. (2018) reported that ratio of trichomes to stomata in cotton is associated with the efficiency of water use. Lei et al. (2018) observed the linkage of the efficiency of water use with variation between veins and stomata under drought stress. In the present study, a strong negative association of RCI% and stomata size with SCY suggested that low RCI% and small stomata are reliable indicators of genotypic heat tolerance response. This analysis indicated that simultaneous selection of high SCY and heat tolerance would be effective in the presence of heat stress. However, density and length of trichomes on the leaf surface, which protect against excessive radiation and reduce the effect of high temperature, were found non-significant, and these results are in contradiction with Celka et al. (2006) and Passos et al. (2009) who emphasized that trichomes play an important role in reducing biotic and abiotic effects. Another study was conducted for evaluating the heat tolerance and stability in multienvironments. The results showed that all selected genotypes were high yielding and stable as compared with the susceptible check and may be tested over all environments to attain an additional stable performance. These findings are in confirmation with some previous reports (Killi and Harem, 2006; Khan et al., 2007; Riaz et al., 2013), which observed the stability and G × E interaction in genotypes of cotton and the effect on SCY. The ability to the combination of some heat-tolerant genotypes was also estimated by a line × tester method among nine hybrids along with their 3 testers (i.e., male) and 3 lines (i.e., females). Genotypes CRIS-342 and NIA-Perkh were observed as best general combiners for SCY with negative GCA effects for RCI%. This finding is in confirmation with the reports of Jatoi et al. (2010) and Srinivas et al. (2014). Five hybrids showed positive SCA and negative heterotic effects for RCI% and also found lowest for HSI. This is in accordance with some previous studies of Abro et al. (2009) and Nidagundi et al. (2012). These findings suggested that the expression of RCI% under field regimes was predominantly controlled by combining ability because of additive genetic variability. Relative contribution of lines or testers individually as compared with lines × testers was also high for RCI and SCY under both temperature regimes, depicting a comparatively significant role of additive genetic variability in the expression of RCI and SCY. In cotton heat tolerance breeding programs, these results are important for selection point of view from segregating populations (Baloch and Bhutto, 2003). In the present study, RCI% and SCY/plant also displayed higher estimates of heritability and genetic advance, indicating the heritability due to additive gene effects and chances of effective selection. Alike degree of heritability and genetic advance were also got by Khan (2002) and Ahsan et al. (2015). The combination of high heritability and genetic advance provides clear picture of the trait in the selection process. The results for additive gene action are in confirmation with the results of Kumaresan et al. (2000) and Larik et al. (2000).

## Conclusion

Selection criteria involving CMT and stomata size concluded to be an effective strategy for the screening of heat-tolerant cotton. Low RCI% and small stomata are good indicators of heat tolerance in optimum as well as heat stress environment. These traits have a good association with SCY. The line × tester analysis revealed two best general combiners for SCY with negative GCA effects for RCI% and five hybrids with positive SCA and heterotic effects for studied traits. Vigilant and rigorous selection of the developed segregating material recognized the heat-tolerant progenies with improved reproductive traits. The identified heat tolerant and wide adaptive germplasm can be further advanced for the release of commercial heat-tolerant cotton varieties.

## Data Availability Statement

The original contributions presented in the study are included in the article/Supplementary Material, further inquiries can be directed to the corresponding author.

## Author Contributions

SaA conceived the idea, executed experiments, recorded the data in the field and laboratory, and prepared the draft manuscript. MR helped in the execution of field and lab experiments, data recording, and analysis and was a major contributor in writing and finalization of the manuscript. ZD helped in execution of field experiments including multilocation trials. ShA performed the data analysis. MS validated the idea, supervised the work, and provided technical inputs in the execution of field experiments. All authors have read and approved the manuscript.

## Conflict of Interest

The authors declare that the research was conducted in the absence of any commercial or financial relationships that could be construed as a potential conflict of interest.

## Publisher’s Note

All claims expressed in this article are solely those of the authors and do not necessarily represent those of their affiliated organizations, or those of the publisher, the editors and the reviewers. Any product that may be evaluated in this article, or claim that may be made by its manufacturer, is not guaranteed or endorsed by the publisher.
