# Supplementary material for: Identification of Heat Tolerant Cotton Lines Showing Genetic Variation in Cell Membrane Thermostability, Stomata, and Trichome Size and Its Effect on Yield and Fiber Quality Traits
Source: Front Plant Sci. 2022 Jan 5;12:804315. doi: 10.3389/fpls.2021.804315 (PMC8766333; doi:10.3389/fpls.2021.804315)
Supplement: Supplementary file 1 [file Table_1.DOCX]

Supplementary Table S1. History/Origin of advance lines and varieties selected for present study

| **Genotypes** | **Origin** | **pedigree/parentage** |
| --- | --- | --- |
| NIA-80 | Indirect mutagenesis | [F_1_ (Chandi-95 × CRIS-9] gamma rays 250 |
| NIA-81 | Indirect mutagenesis | [F_1_ (Sadori x NIAB-111] gamma rays 250 Gy |
| NIA-83 | Mutant | CRIS-134 gamma rays 250 Gy |
| NIA-84 | Mutant | DEM-134 gamma rays 200 Gy |
| NIA-85 | Mutant | DEM-84 gamma rays 250 Gy |
| NIA-86 | Mutant | DEM-84 gamma rays 200 Gy |
| NIA-M-30 | Mutant | Sadori/gamma rays 250 Gy |
| NIA-HM-327 | Indirect mutagenesis | F_1_(Sohni × DPL-14) gamma rays 250 Gy |
| NIA-HM-329 | Indirect mutagenesis | [F_1(_NIA-Ufaq × Chandi-95) gamma rays 250 Gy |
| NIA-HM-335 | Indirect mutagenesis | [F_1(_NIA-Ufaq × Chandi-95) gamma rays 300 Gy |
| NIA-H-1 | Hybridization | Sohni × Chandi-95 |
| NIA-H-24 | Hybridization | Sohni × NIAB-78 |
| NIA-M31 | Mutant | Sohni/ gamma rays 250 Gy |
| NIA-M32 | Mutant | NIA-Noori/gamma rays 250 Gy |
| NIA-M33 | Mutant | Sadori/gamma rays 200 Gy |
| NIA-M34 | Mutant | Sohni/gamma rays 250 Gy |
| NIA-Perkh | Mutant | Sarmast gamma rays 200 Gy ^60^ Co source |
| NIA-H-13 | Hybridization | NIA-Ufaq × Sohni |
| NIA-HM-2 | Indirect mutagenesis | [F_1(_NIA-Ufaq × Sadori) gamma rays 250 Gy |
| NIA-HM-48 | Indirect mutagenesis | [F_1_ (Chandi-95 x CRIS-134] gamma rays 250 Gy |
| NIA-M-2 | Mutant | Sadori/gamma rays 250 Gy |
| NIA-HM-1 | Indirect mutagenesis | [F_1_ (Chandi-95 x CRIS-134] gamma rays 300 Gy |
| NIA-H-36 | Hybridization | NIAB-111 × Chandi-95 |
| NIA-H-12 | Hybridization | NIAB-111 × Sadori |
| NIA-Bt-1 | Hybridization | FH-1000 ×Coker312 |
| NIA-Bt-2 | Hybridization | Sadori × IR3701 |
| NIA-Bt-3 | Hybridization | NIAB-78 × FH-1000 |
| NIA-Bt-4 | Hybridization | NIAB-78 × FH-901 |
| NIA-Bt-5 | Hybridization | NIAB-78 × CRIS-542 |
| NIA-HM-320 | Indirect mutagenesis | [F_1_ (Chandi-95 × CRIS-542] gamma rays 200 Gy |
| NIA-HM-322 | Indirect mutagenesis | [F_1_ (Chandi-95 × CRIS-342] gamma rays 250 Gy |
| NIA-HM-323 | Indirect mutagenesis | [F_1_ (Chandi-95 × FH-1000] gamma rays 300 Gy |
| NIA-HM-337 | Indirect mutagenesis | [F_1_ (Sadori × CRIS-542] gamma rays 200 Gy |
| NIA-HM-338 | Indirect mutagenesis | [F_1_ (Sadori × CRIS-342] gamma rays 250 Gy |
| NIA-HM-321 | Indirect mutagenesis | [F_1_ (Sadori × FH-1000] gamma rays 300 Gy |
| NIA-H-31 | Hybridization | NIAB-78 ×Sohni |
| NIA-H-32 | Hybridization | Sohni × NIAB-111 |
| NIA-H-01 | Hybridization | NIA-Noori × NIAB-78 |
| NIA-Okra-24 | Hybridization | La okra 541, HRVO × Sadori |
| NIA-H-29 | Hybridization | Sohni × DPL-14 |
| NIA-H-30 | Hybridization | NIA-Ufaq × DPL-14 |
| NIA-HM-311 | Indirect mutagenesis | [F_1_ (Sohni × Sadori] gamma rays 250 Gy |
| NIA-H-67 | Hybridization | NIAB-78 × DPL-14 |
| CRIS-121 | Hybridization | - |
| CRIS-9 | Hybridization | - |
| Sindh-1 | Hybridization | - |
| Hari Dost | Hybridization | - |
| Sohni | Mutant | NIAB-78/ gamma rays 300 Gy |
| NIA-Ufaq | Mutant | DEM-84/ gamma rays 250 Gy |
| CRIS-342 | Hybridization | CRIS-52 × CRIS-121 |
| Chandi-95 | Mutant | NIAB-78/ gamma rays 250 Gy |
| Sadori | Indirect mutagenesis | F_1_(Shaheen x DPL-14) gamma rays 250 Gy |
| Shahbaz | Hybridization | - |
| IR-3701 | Hybridization | - |
| NIAB-78 | Mutant | - |
| NIAB-111 | Indirect mutagenesis | F_1_ (NIAB 313/12 x CIM 1000) gamma rays 300 Gy |
| CIM-469 | Hybridization | CIM425 ×755-6/93 |
| CRIS-134 | Hybridization | NIAB-78 ×DPL70 |

Supplementary Table S2. Mean squares from the analysis of variance of different traits among 58 diverse cotton genotypes

| Source | DF | RCI% | No. of bolls/plant | Seed cotton yield (kg/ha) | Staple length (mm) | GOT (%) |
| --- | --- | --- | --- | --- | --- | --- |
| Replicates | 2 | 244.6 | 2186.05 | 0.7 | 228.4 | 286.35 |
| Genotypes | 57 | 755.0** | 785596.0** | 80.8** | 187.7 | 270.30 |
| Sowing dates | 1 | 360.1* | 1.4508** | 1996.8** | 63.1 * | 1731.1* |
| Sowing dates × Genotypes | 57 | 70.8* | 136227** | 17.0** | 188.9 | 284.72 |
| Error | 230 | 59.7 | 3668.50 | 3.45 | 187.9 | 288.10 |

*,** Significance levels at 5% and 1% respectively.

Supplementary Table S3. Mean squares from the analysis of variance of stomata and trichome size of selected 18 cotton genotypes

| Source | DF | Stomata Size | Trichome Size |
| --- | --- | --- | --- |
| Replicates | 2 | 15.62 | 389515 |
| Genotypes | 17 | 1614.43** | 55740* |
| Error | 34 | 4.04 | 16035 |

*,** Significance levels at 5% and 1% respectively.

Supplementary Table S4. Combined ANOVA for seed cotton yield of 18 selected cotton genotypes tested over different environments

| Sources of Variation | Degree of Freedom | Sum of Squares | Mean Squares | F value |
| --- | --- | --- | --- | --- |
| Replicates (R) | 3 | 0.0372 | 0.0124 |  |
| Environment (E) | 4 | 16.422 | 4.1055 | 1173.43** |
| Genotypes (G) | 17 | 15.396 | 0.9056 | 258.86** |
| G × E Interaction | 68 | 5.674 | 0.0834 | 23.85** |
| Error | 267 | 0.934 | 0.0035 |  |
| Total | 359 | 38.464 |  |  |

Supplementary Table S5. Mean squares from ANOVA of RCI % (field and glasshouse) and SCY (field) of cotton genotypes using line × tester analysis in F_1_ generation

| Sources of Variation | DF | RCI % (Glasshouse) | RCI % (Field) | SCY (g/plant) |
| --- | --- | --- | --- | --- |
| Replicates | 2 | 2.19 | 1.70 | 112.25 |
| Temperature regimes (G) | 1 | 12.9* | 0.40** | 2600.5** |
| Temperature regimes (P) | 1 | 42.2** | 4.41* | 30.64** |
| Regimes (R) (C) | 1 | 4.08* | 1.19^ns^ | 24.65** |
| Genotypes | 14 | 916.5** | 491.2** | 74.30** |
| Parents | 5 | 799.8** | 681.2** | 32.35** |
| Parents vs. Crosses | 1 | 984.8* | 1197** | 1404.9** |
| Crosses | 8 | 996.3** | 306.2* | 35.44** |
| Lines | 2 | 33.1* | 2.44^ns^ | 2.74^ns^ |
| Testers | 2 | 272.9** | 34.53** | 11.54** |
| Lines × Testers | 4 | 306.5** | 343.2** | 41.26** |
| Genotypes × Temperature regimes | 14 | 1.7* | 3.12** | 2.62** |
| Parents × Temperature regimes | 5 | 9.18** | 3.94** | 4.36** |
| Parents vs. Crosses × Temperature regimes | 1 | 8.3* | 4.49* | 4.05* |
| Crosses × Temperature regimes | 8 | 16.7^ns^ | 2.80* | 1.14^ns^ |
| Lines × Temperature regimes | 4 | 0.47^ns^ | 0.38^ns^ | 1.14^ns^ |
| Testers × Temperature regimes | 2 | 0.30* | 0.47^ns^ | 3.93* |
| Lines × Testers × Temperature regimes | 4 | 2.60* | 3.41* | 4.23** |
| Error mean | 58 | 8.625 | 1.67 | 48.18 |

*,** significant levels at 5% and 1% respectively and ns = non-significant
